# Chest tube fixation strength depends on suture caliber and fixation technique rather than tube diameter: a biomechanical cadaver study

**DOI:** 10.1007/s00068-026-03369-0

**Published:** 2026-10-26

**Authors:** Johann Justus Fricke, Tobias Schöbel, Ric Meißner, Stefan Schleifenbaum, Christian Kleber, Isabella Metelmann, Sebastian Krämer

**Affiliations:** 1 Department of Orthopedic, Trauma, and Plastic Surgery, University of Leipzig, Liebigstraße 20, 04103 Leipzig, Germany; 2 ZESBO – Center for Research on Musculoskeletal Systems, Semmelweisstraße 14, 04103 Leipzig, Germany; 3 Fraunhofer Institute for Machine Tools and Forming Technology, Nöthnitzer Straße 44, 01187 Dresden, Germany; 4 Department of Visceral, Transplantation, Thoracic and Vascular Surgery, Leipzig University, Liebigstraße 20, 04013 Leipzig, Germany

**Keywords:** Chest tubes, Thoracic injuries, Suture, Biomechanics, Fixation techniques, Trauma, Emergency, Combat care

## Abstract

**Background:**

Chest tube dislodgement remains a relevant complication in emergency, trauma, and intensive care medicine. In a previous biomechanical study, the Modified Johannesburg technique using Mersilene USP 1 demonstrated superior fixation strength compared with other chest tube fixation techniques. The present study aimed to determine whether suture size, suture material, and chest tube diameter influence fixation strength.

**Methods:**

A prospective interventional biomechanical cadaveric study was performed using fresh porcine thoracic wall specimens. A total of 320 load-to-failure tests were conducted under standardized laboratory conditions. Two fixation techniques, Modified Johannesburg and Purse String, were compared using two chest tube diameters (20/28 Ch) and four non-absorbable braided suture configurations: Mersilene USP 1, Mersilene USP 2 − 0, Silk USP 1, and Silk USP 2 − 0. Each experimental group consisted of 20 specimens. Specimens were tested in a uniaxial testing machine until construct failure. Force [N] and elongation [mm] at failure were measured. The experiments were videotaped, and the failure mechanism was analyzed.

**Results:**

The Modified Johannesburg technique demonstrated significantly higher pull-out forces compared to the Purse String technique (125.0 vs. 66.4 N; *p* < 0.001), as did larger suture sizes (USP 1 vs. 2 − 0; *p* < 0.001) and Mersilene compared to Silk (114.6 vs. 76.9 N; *p* < 0.001). Chest tube size had only a very small and inconsistent effect on pull-out strength. The predominant failure mode was suture rupture (94.7%).

**Conclusions:**

Chest tube fixation strength is significantly influenced by fixation technique, suture size, and suture material. The effect of chest tube diameter on fixation strength appeared negligible. The Modified Johannesburg technique, size 1 sutures, and Mersilene material provided superior biomechanical stability. Since failure occurred predominantly by suture rupture, the suture appears to represent the weakest link of the fixation construct.

## Introduction

Thoracic trauma remains a frequent and potentially life-threatening condition in both civilian and military emergency care. In particular, untreated tension pneumothorax is considered a preventable cause of traumatic death, and tube thoracostomy remains a standard intervention for rapid pleural decompression and stabilization in patients with pneumothorax or hemothorax [1–4]. Although chest tube placement is a routine procedure, it is not free of complications [5]. Reported complication rates vary considerably and include insertional, positional, removal-related, infectious/immunologic, and instructional/educational/equipment-related complications [6, 7], with overall complication rates of approximately 20% in meta-analyses [8] and up to 40% in some series [9]. Among these complications, unintended chest tube dislodgement is particularly relevant, as it may require repeated intervention, prolong hospitalization, or lead to recurrent pneumothorax or hemorrhage [10].

For this reason, secure fixation of chest tubes is a key component of chest tube management. Current procedural recommendations emphasize that chest tubes should be fixed to prevent accidental fall-out, but they provide limited evidence-based guidance on the optimal suture technique, suture material or suture size [11–13]. Several fixation methods have been described, including the Purse String, Roman Sandal and Modified Johannesburg techniques [1, 14–16]. However, the mechanical performance of these techniques has only recently become the subject of systematic biomechanical investigation.

In a previous standardized porcine thoracic wall model, we demonstrated that the Modified Johannesburg technique provides superior biomechanical stability compared with Roman Sandal and Purse String fixation when using Mersilene USP 1. This superiority was observed across different chest tube sizes and tube materials, supporting the Modified Johannesburg technique as the preferred fixation method in settings where high traction forces may occur [1]. Importantly, the predominant failure mechanism in that study was suture rupture, followed by tube rupture, knot loosening, and skin failure [1]. These findings suggest that, once an optimal fixation geometry has been selected, further improvements in fixation strength may depend less on the overall suture configuration and more on the mechanical properties of the fixation construct itself. From a biomechanical perspective, the stability of a chest tube fixation construct is likely determined by the interaction between skin, suture, knot, and tube surface. The Modified Johannesburg technique may be particularly strong because it distributes traction over multiple anchoring points and increases the contact area between suture and tube. In such a construct, higher resistance to displacement may result from both intrinsic suture strength and frictional resistance at the suture–tube interface. This provides a plausible mechanical rationale that larger tube diameters could increase fixation strength by increasing contact surface and friction, while thicker sutures may resist rupture better than thinner sutures. Conversely, differences in material properties may also be relevant: braided polyester and Silk sutures differ in tensile strength, elongation, surface structure, handling characteristics, and knot behavior, all of which may influence construct stability under axial traction [17–20].

This question is clinically relevant because chest tube size and suture selection vary widely in daily practice. Smaller tubes may be preferred in selected indications because they are associated with less tissue trauma and pain, whereas larger tubes are still commonly used in trauma, hemothorax, or high-viscosity pleural collections [9, 21, 22]. General suture studies show that knot security and tensile performance are influenced by suture material, diameter, knot configuration, and number of throws [17–19]. However, whether these material-specific differences translate into clinically relevant differences in chest tube fixation strength remains unclear.

The present study therefore builds directly on our previous findings by investigating whether the biomechanical superiority of the Modified Johannesburg technique is affected by changes in chest tube size, suture diameter, and suture material. Using the same standardized experimental setting, we compared the Modified Johannesburg and Purse String techniques with two chest tube sizes, 20 Ch and 28 Ch, and four non-absorbable braided suture configurations: Mersilene USP 1, Mersilene USP 2 − 0, Silk USP 1, and Silk USP 2 − 0. The aim of this study was to determine whether fixation strength is modified by suture size, suture material, and/or tube size within otherwise comparable fixation constructs. We hypothesized that larger tube diameters and larger suture calibers would increase resistance to displacement, potentially due to greater frictional contact and higher tensile reserve, and that polyester sutures would provide greater fixation strength than Silk sutures of corresponding size.

## Methods

### Study design

This prospective interventional biomechanical cadaveric study evaluated the influence of suture size, suture material and chest tube size on the fixation strength of two chest tube fixation techniques using porcine thoracic wall specimens. A total of 320 experiments were conducted under standardized laboratory conditions. Sample size was based on the standardized experimental design and effect sizes observed in our previous biomechanical investigation using the same porcine thoracic wall model [1]. Given the balanced factorial design with 20 specimens per group, the study was considered sufficiently powered to detect clinically and biomechanically relevant differences between fixation techniques and suture configurations.

### Specimens and preparation

Fresh porcine thoracic wall specimens were obtained one day prior to testing. Until preparation and testing, specimens were stored under refrigeration at 4 °C. Rectangular sections measuring 10 × 6 cm, with an average thickness of approximately 1.5 cm, were prepared from each thoracic wall using a standardized, 3D-printed template. A central perforation was created for chest tube insertion. Each specimen was marked at 5 cm: the lower mark defined the clamp position within the testing device, whereas the upper mark indicated the tube insertion site.

### Experimental groups

**Fig. 1:**
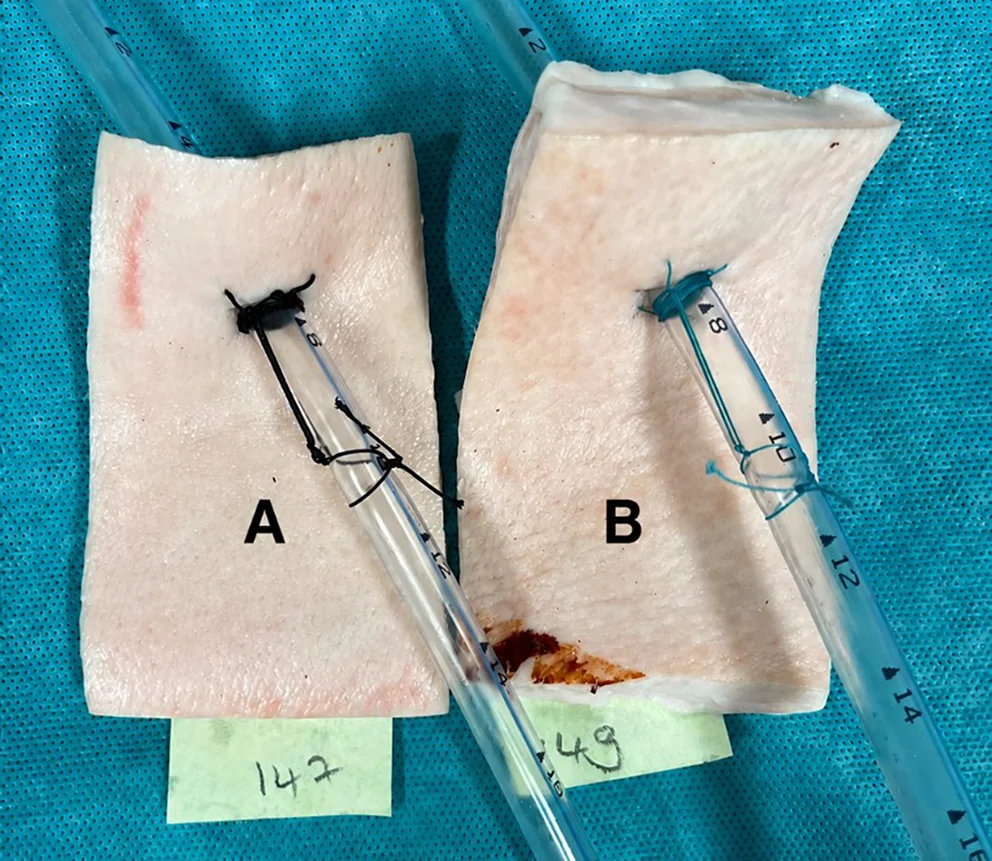
Two specimens with 28 Ch PVC tubes, fixed using the Modified Johannesburg technique with Silk (**A**) or Mersilene (**B**). PVC: Polyvinyl chloride

A total of 320 tests were performed across 16 experimental groups. Groups were defined by fixation technique, chest tube size, suture size, and suture material. Two fixation techniques were tested: Purse String and Modified Johannesburg (Fig. 1). Two chest tube sizes were used: 20 Ch and 28 Ch. Four non-absorbable braided suture configurations were evaluated: Mersilene USP 1, Mersilene USP 2 − 0, Silk USP 1, and Silk USP 2 − 0. Braided sutures were deliberately selected because they are commonly used for chest tube fixation in clinical trauma and thoracic surgery practice and provide high knot security and frictional stability. Monofilament sutures were not included in the present study in order to limit experimental complexity and allow focused comparison of clinically established braided materials. Each group of tested sutures consisted of 20 specimens. (Table 1).

**Table 1:** Configuration of the 16 groups, including fixation technique, chest tube size (Ch), suture material, and suture size (USP). USP: United States Pharmacopeia

Fixation technique	Chest tube size (Ch)	Suture material and Suture size (USP)
Modified Johannesburg (JO)	20, 28	Mersilene 1 | Mersilene 2 − 0 | Silk 1 | Silk 2 − 0
Purse String (PS)	20, 28	Mersilene 1 | Mersilene 2 − 0 | Silk 1 | Silk 2 − 0

Fixations were performed by two experienced thoracic surgeons using standardized technique-specific suture configurations. The tested sutures were non-absorbable braided Mersilene USP 1, Mersilene USP 2 − 0, Silk USP 1, and Silk USP 2 − 0. PVC chest tubes of 20 Ch and 28 Ch were used for all corresponding groups. Specimens were labeled with alphanumeric codes representing fixation technique, suture size, suture material, and chest tube size. The complete set of specimen codes was randomized using the randomization service provided by random.org (Randomness and Integrity Services Ltd., Dublin, Ireland).

### Testing protocol

**Fig. 2:**
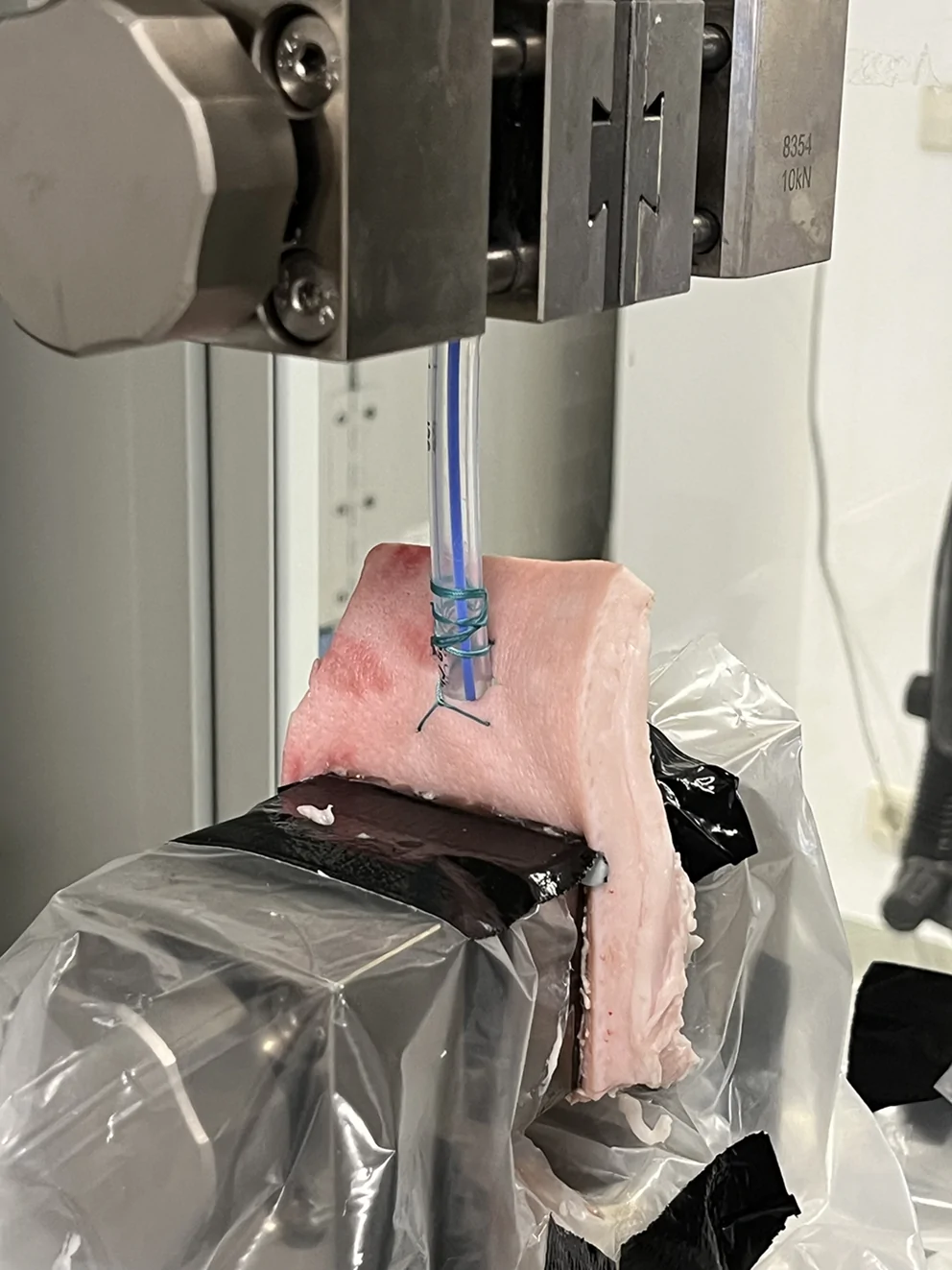
Experimental setup showing the uniaxial testing machine with a specimen

Specimens were mounted in the lower clamps of a uniaxial testing machine (Allroundline Z10, Zwick/Roell GmbH & Co. KG, Ulm, Germany) with a 2.5 kN load cell [1]. The inserted tube was secured at a 7 cm distance from the upper clamp (Fig. 2). A frontal camera (Canon EOS 70D; Canon Inc., Tokyo, Japan) was used for documentation. A vertical load-to-failure test was performed under standardized conditions. Force (N) and crosshead travel (mm) were recorded continuously until construct failure. Prior to testing, a preload of 5 N was applied. Testing was performed at the maximum achievable crosshead speed of 1750 mm/min. The maximum force recorded before failure was defined as the load-to-failure. The failure mode was documented for each experiment and categorized according to the observed mechanism, including suture rupture, tube rupture, knot loosening, skin failure, or other failure patterns.

### Statistical analysis

Statistical analyses were performed using R (version 4.5.1; R Core Team, 2025, Vienna, Austria) within the RStudio environment (RStudio Team, version 2026.04.0 + 526; Boston, MA). Prior to analysis, assumptions of normality and homogeneity of variances were assessed. Normality was evaluated using the Shapiro–Wilk test and visual inspection of Q–Q plots and histograms. Homogeneity of variances was assessed using Levene’s test. A four-way factorial ANOVA based on a linear model with Type III sums of squares was performed to evaluate the effects of suture technique, suture size, suture material, and chest tube size on pull-out force and elongation. Given the balanced design, the analysis was considered robust to violations of assumptions. Additionally, heteroskedasticity-robust standard errors (HC3) and log-transformed sensitivity analyses were applied. Effect sizes were calculated as partial eta squared (η²p) and classified following Cohen’s conventions (small: 0.01, medium: 0.06, large: ≥ 0.14). Post-hoc comparisons were performed using estimated marginal means with pairwise contrasts. A significance level of *p* < 0.05 was considered statistically significant. Data visualization was performed using R and the *ggplot2* package, with final figure editing conducted in *Inkscape* (version 1.4.2; Inkscape Project, 2025).

## Results

### Effect of suture technique

**Fig. 3:**
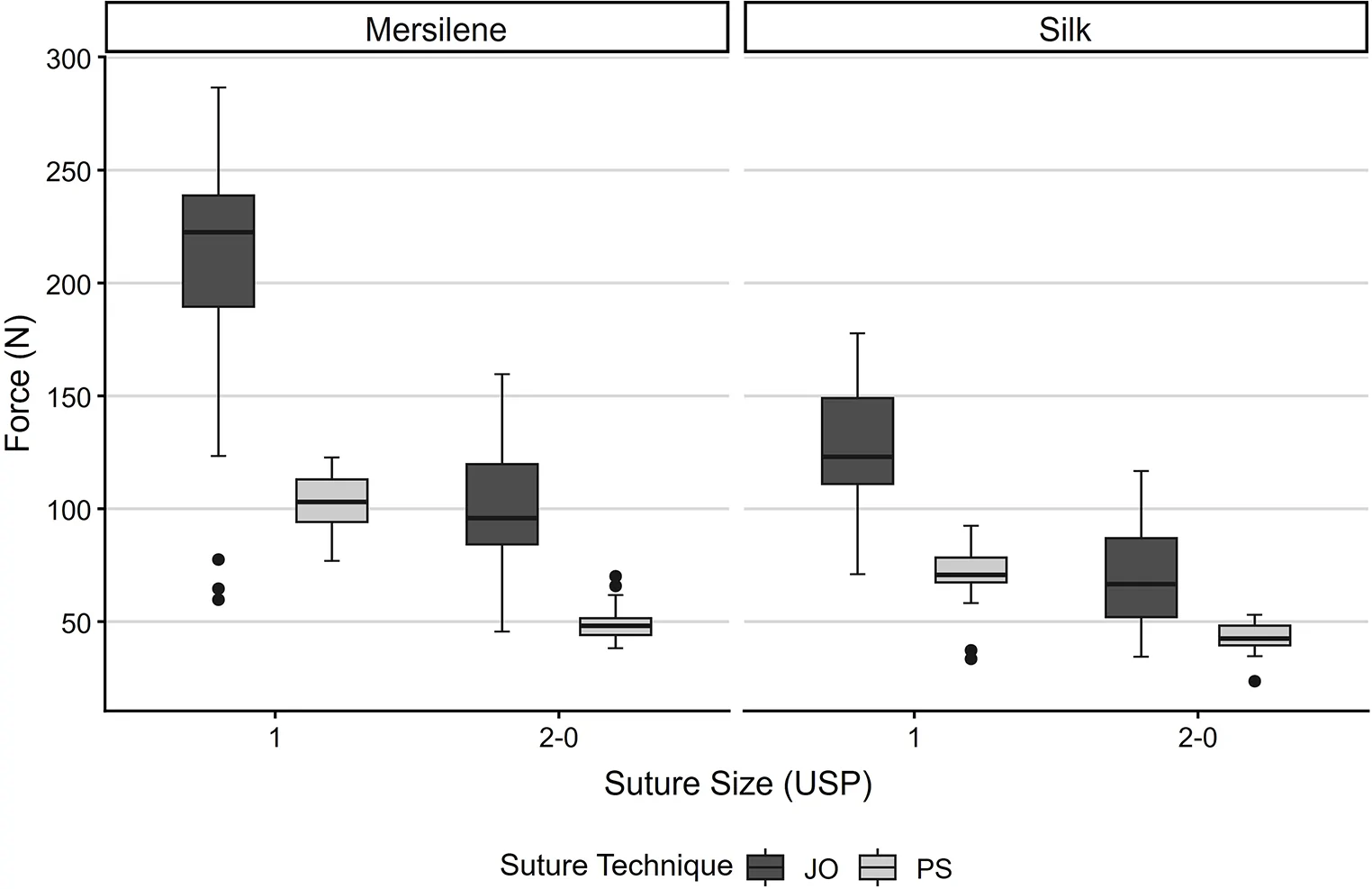
Boxplots of breaking forces in Newton (N) of two different suture techniques (JO and PS) across two different suture materials (Mersilene and Silk) and two different suture sizes (USP 1 and 2 − 0). JO: Modified Johannesburg technique, PS: Purse String technique, USP: United States Pharmacopeia

Suture technique had a significant effect on pull-out force, with a large partial effect size (*F (1*,*304)* = 409.49, *p* < 0.001, η²*p* = 0.57; Fig. 3). Overall, the Modified Johannesburg technique resulted in substantially higher pull-out forces compared with the Purse String technique (estimated marginal means: 125.0 N vs. 66.4 N, *p* < 0.001). Significant interaction effects were observed between suture technique and other experimental factors, including suture material (F(1,304) = 42.91, *p* < 0.001, η²*p* = 0.12) and suture size (F(1,304) = 47.79, *p* < 0.001, η²*p* = 0.14). In addition, a small but statistically significant three-way interaction among technique, suture material, and suture size was detected (*F*(1,304) = 4.69, *p* = 0.031, η²*p* = 0.02). These findings indicate that fixation strength depends on the interaction between fixation technique and suture characteristics. Even after accounting for unequal variances, the effects of suture technique remain stable, confirming the robustness of the finding.

### Effect of suture size

Suture size showed one of the strongest effects on pull-out force (*F(1*,*304)* = 441.84, *p* < 0.001, η²*p* = 0.59; Fig. 3). Larger sutures (USP 1) were associated with significantly higher pull-out forces compared to smaller sutures (USP 2 − 0), with a mean difference of 60.9 N (*p* < 0.001). Importantly, suture size was involved in multiple significant interaction effects, including interactions with suture technique (F(1,304) = 47.79, *p* < 0.001, η²*p* = 0.14) and suture material (F(1,304) = 44.19, *p* < 0.001, η²*p* = 0.13).

### Effect of suture material

Suture material significantly influenced pull-out forces (*F*(1,304) = 169.25, *p* < 0.001), η²*p* = 0.36; Fig. 3). Mersilene sutures demonstrated higher pull-out strength compared to Silk sutures (114.6 N vs. 76.9 N, *p* < 0.001). In addition, suture material significantly interacted with suture technique (F(1,304) = 42.91, *p* < 0.001, η²*p* = 0.12) and suture size (F(1,304) = 44.19, *p* < 0.001, η²*p* = 0.13), indicating that the mechanical performance of the material is context-dependent.

### Effect of chest tube size

Chest tube size did not show a statistically significant effect on pull-out force in the primary analysis (*F*(1,304) = 2.55, *p* = 0.112, η²*p* = 0.008). Estimated marginal means were similar between the two tube sizes investigated (20 Ch: 93.4 N vs. 28 Ch: 98.0 N, mean difference: 4.6 N, *p* = 0.112). Likewise, no significant two-way or three-way interactions involving tube size were detected. In the sensitivity analysis using log-transformed force values, chest tube size approached statistical significance (*F*(1,304) = 3.72, *p* = 0.055, η²*p* = 0.01), while the four-way interaction among technique, suture material, suture size, and chest tube size reached borderline significance (F(1,304) = 3.88, *p* = 0.050, η²*p* = 0.01). Nevertheless, effect sizes for tube-related factors remained small, indicating limited clinical relevance.

### Elongation

Greater elongation at failure was significantly associated with the Modified Johannesburg technique, USP 1 sutures, Mersilene, and 20 Ch chest tubes (all *p* < 0.001), including interactions among these parameters. After adjusting for force, differences in elongation between groups were markedly reduced, while the overall pattern of effects remained.

### Mechanism of failure

The dominant failure mechanism was suture rupture, accounting for 94.7% of all cases. All other failure modes occurred only rarely (Fig. 3). This finding suggests that the suture is the limiting factor in the fixation construct of chest tubes.

**Fig. 4:**
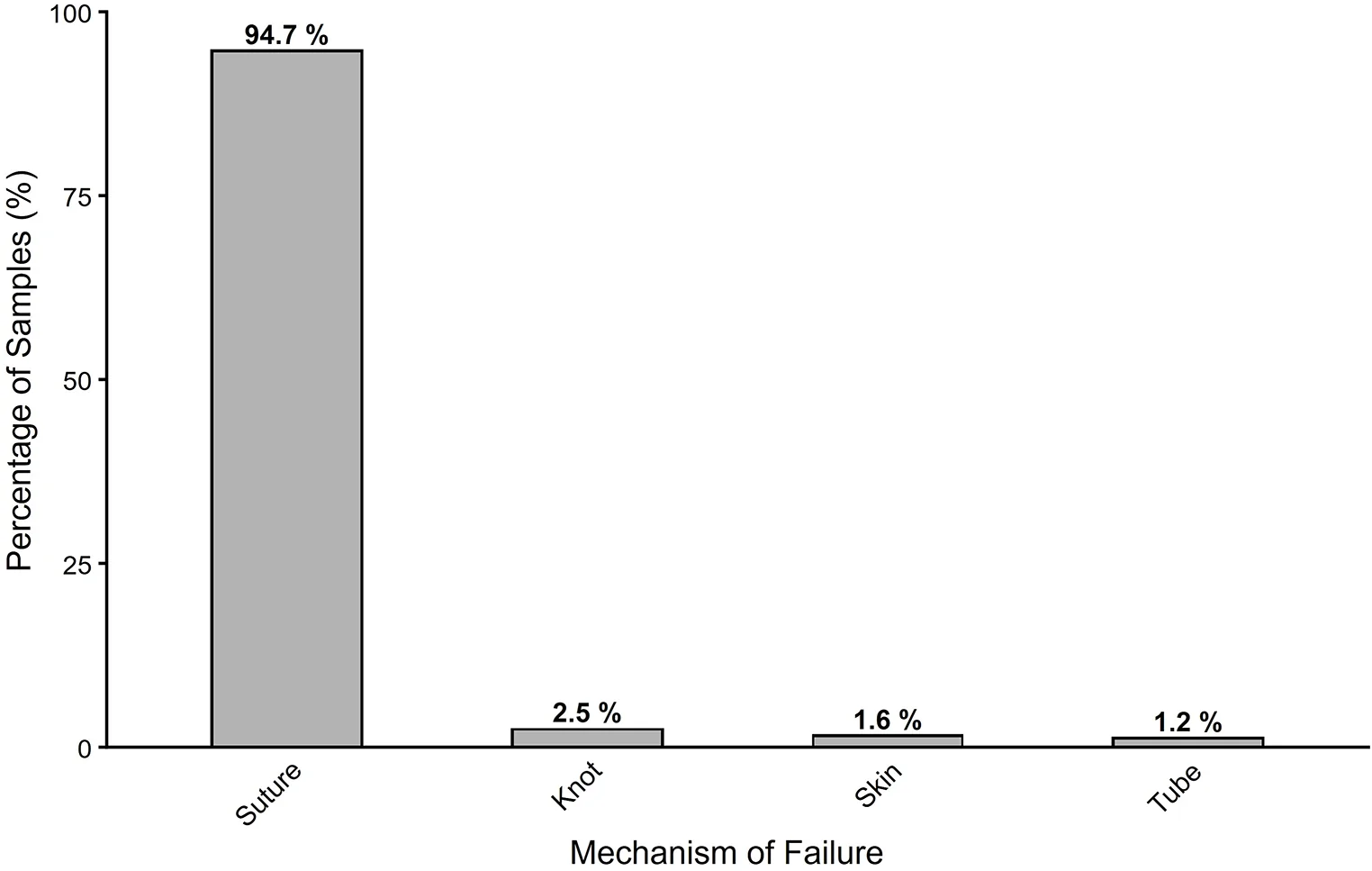
Mechanism of failure in percent (%) of all 320 samples. Suture: rupture of the suture; Knot: loosening of the knot; Skin: rupture of the pig skin; Tube: rupture of the chest tube

## Discussion

The present study investigated whether chest tube fixation strength is influenced by suture size, suture material, and/or chest tube size in a standardized porcine thoracic wall model. This study directly builds on our previous work, in which the Modified Johannesburg technique using Mersilene USP 1 demonstrated superior biomechanical stability compared with the Roman Sandal and Purse String technique across different tube sizes and tube materials [1]. In the present study, we confirmed the biomechanical superiority of the Modified Johannesburg technique over the Purse String technique and further demonstrated that suture size and suture material significantly affect fixation strength, whereas chest tube size did not. In practical terms, the strongest constructs were achieved by combining the Modified Johannesburg technique with thicker USP 1 sutures and Mersilene.

The most frequent failure mechanism in the present study was suture rupture, occurring in 303 of 320 tests. This finding is consistent with our previous biomechanical study, in which failure of the suture was also the predominant failure mode [1]. It suggests that, under axial traction, the suture itself represents the critical limiting component of the fixation construct. Accordingly, once an effective fixation geometry has been chosen, the mechanical properties of the suture appear to become decisive for overall construct stability.

The significant influence of suture size supports this interpretation. USP 1 sutures demonstrated substantially higher load-to-failure values than USP 2 − 0 sutures. This observation is consistent with established biomechanical principles, as larger-caliber sutures generally tolerate greater tensile loads before rupture. General surgical literature on knot security and suture mechanics confirms that suture gauge, material properties, knot configuration, number of throws, and pulling direction all influence knot performance and mechanical stability [18, 19]. In the present model, the large effect size observed for suture caliber indicates that using a thinner suture may markedly reduce fixation strength, even when the same fixation technique is applied.

Suture material also significantly affected fixation strength. Mersilene demonstrated higher load-to-failure values than Silk. This is consistent with experimental studies showing that tensile and knot performance differ between suture materials, including polyester and Silk [18, 20]. Although Silk is widely used because of its favorable handling and knotting characteristics [23], braided polyester sutures such as Mersilene may provide superior resistance to traction forces in this setting. Given that accidental traction can occur during patient transfer, mobilization, intensive care treatment, or prehospital care, this difference may be clinically relevant. Although the exact traction forces acting on chest tubes in clinical practice are difficult to quantify and likely vary substantially depending on patient movement, transport conditions, and accidental manipulation, the observed differences in load-to-failure may still be clinically meaningful. In a recent scoping review, Wong et al. reported experimental failure forces ranging from approximately 19 to 180 N across different chest drain fixation methods and cited an average peak human pulling force of approximately 150 N [10]. Against this background, a fixation construct tolerating approximately 120 N before failure provides a substantially larger mechanical safety reserve than a construct failing at approximately 60 N. Particularly in trauma care, intensive care transport, or prehospital settings, where sudden unintentional traction events may occur, increased fixation strength may reduce the risk of accidental tube dislodgement and the need for repeated thoracic intervention.

In contrast to our initial assumption, chest tube diameter did not significantly influence fixation strength. We hypothesized that larger chest tube sizes might increase the contact area between tube and suture and thereby improve frictional resistance. However, the influence of chest tube size on pull-out forces was inconsistently significant, and if so, with only very small effect sizes, suggesting a negligible effect with limited clinical relevance. This result is in line with our previous study, in which the Modified Johannesburg technique showed superior stability irrespective of tube size and tube material [1]. The present data suggest that any potential increase in friction caused by a larger tube diameter is outweighed by the tensile properties of the suture. Since most constructs failed by suture rupture, the system appears to reach the mechanical limit of the suture before tube diameter can provide a relevant stabilizing effect.

This finding also fits into the broader clinical discussion regarding chest tube size. Several studies have questioned whether larger chest tubes are necessarily superior for clinical drainage outcomes in trauma patients [21, 22]. While the clinical indication remains decisive for selecting chest tube size, the present biomechanical data suggest that tube size should not be expected to improve fixation strength. Instead, fixation stability appears to depend primarily on the fixation technique and the chosen suture [24].

The superiority of the Modified Johannesburg technique over the Purse String technique confirms and extends previous findings. Ringel et al. demonstrated that the Modified Johannesburg technique provides greater fixation strength than several alternative methods, including the Purse String technique, in a porcine model [15]. Our previous study further supported the Modified Johannesburg technique as the most stable construct compared with Roman Sandal and Purse String techniques [1]. The present results show that this superiority persists when different suture sizes and suture materials are used. This suggests that the geometry of the Modified Johannesburg technique provides a mechanically favorable distribution of traction forces at the skin–suture–tube interface.

Several limitations must be considered. First, this was an ex vivo biomechanical study using porcine thoracic wall specimens. Although porcine tissue provides a standardized and established model for mechanical testing of chest tube fixation techniques, it cannot fully reproduce human tissue properties, wound healing, tissue edema, infection, pain-related movement, or biological changes over time. Second, the study assessed immediate vertical axial load to failure under laboratory conditions. In clinical practice, chest tube dislodgement may result from multidirectional, repetitive, or oblique traction forces. Because the Modified Johannesburg technique relies on multiple anchoring points and circumferential frictional contact between the suture and tube, non-axial loading may alter both force distribution and friction at the suture–tube interface. Whether this would increase or reduce its relative mechanical advantage cannot be inferred from the present axial load-to-failure experiments and should be investigated using dedicated multidirectional loading protocols. Nevertheless, axial traction was deliberately selected as a standardized and reproducible worst-case loading scenario because unintended chest tube dislodgement frequently occurs during patient transfer, mobilization, or accidental pulling events, in which substantial longitudinal traction may be applied to the tube. While cyclic and multidirectional loading protocols may better reflect complex clinical movement patterns, they would also introduce substantially greater experimental variability and were beyond the scope of the present biomechanical comparison study. Third, only two fixation techniques, two tube diameters, and four suture configurations were tested. In addition, only braided non-absorbable sutures were evaluated. Therefore, the present findings cannot be generalized to monofilament materials such as Nylon or Polypropylene, which may differ substantially in knot security, elasticity, and frictional behavior and the results cannot be generalized to all available chest tubes, suture materials, or fixation methods. Future biomechanical studies should directly compare braided and monofilament suture materials under otherwise identical experimental conditions. Furthermore, the individual allocation of procedures between the two surgeons was not prospectively recorded. Therefore, a potential operator effect or an imbalance in operator distribution across experimental groups cannot be excluded, although all fixations were performed by experienced thoracic surgeons using standardized technique-specific configurations. Finally, this study assessed mechanical stability only and did not evaluate clinical outcomes such as pain, insertion-site complications, wound healing, cosmetic results, or actual dislodgement rates in patients.

Despite these limitations, the present study provides relevant biomechanical evidence for chest tube fixation. The results refine our previous findings by showing that fixation strength is not only determined by the fixation technique but also by suture caliber and suture material. In contrast, tube size did not significantly contribute to construct stability. Therefore, when maximum fixation strength is required, the use of the Modified Johannesburg technique in combination with a mechanically robust suture, particularly Mersilene USP 1, appears advantageous.

## Conclusion

Chest tube fixation strength was significantly influenced by fixation technique, suture size, and suture material, but not by chest tube size. The Modified Johannesburg technique, USP 1 sutures, and Mersilene material provided superior biomechanical stability. Since failure occurred predominantly by suture rupture, the suture appears to be the critical limiting component of the fixation construct. These findings may support evidence-based selection of fixation technique and suture material in trauma, emergency, and critical care settings where accidental chest tube dislodgement may have clinically relevant consequences.

## Data Availability

The datasets generated and analyzed during the current study are available from the corresponding author upon reasonable request.

## Abbreviations

Ch: Charrière
LTF: Load-to-failure
PVC: Polyvinyl chloride
3D: Three-dimensional
ROM: Range of motion
USP: United States Pharmacopeia
